# Cigarette Smoke Decreases the Maturation of Lung Myeloid Dendritic Cells

**DOI:** 10.1371/journal.pone.0152737

**Published:** 2016-04-08

**Authors:** Elena Arellano-Orden, Carmen Calero-Acuña, Nicolás Moreno-Mata, Lourdes Gómez-Izquierdo, Verónica Sánchez-López, Cecilia López-Ramírez, Daniela Tobar, José Luis López-Villalobos, Cesar Gutiérrez, Ana Blanco-Orozco, José Luis López-Campos

**Affiliations:** 1 Unidad Médico-Quirúrgica de Enfermedades Respiratorias, Instituto de Biomedicina de Sevilla (IBiS), Hospital Universitario Virgen del Rocío/Universidad de Sevilla, Seville, Spain; 2 CIBER de Enfermedades Respiratorias (CIBERES), Instituto de Salud Carlos III, Madrid, Spain; University Hospital Freiburg, GERMANY

## Abstract

**Background:**

Conflicting data exist on the role of pulmonary dendritic cells (DCs) and their maturation in patients with chronic obstructive pulmonary disease (COPD). Herein, we investigated whether disease severity and smoking status could affect the distribution and maturation of DCs in lung tissues of patients undergoing elective pneumectomy or lobectomy for suspected primary lung cancer.

**Materials and Methods:**

A total of 75 consecutive patients were included. Spirometry testing was used to identify COPD. Lung parenchyma sections anatomically distant from the primary lesion were examined. We used flow cytometry to identify different DCs subtypes—including BDCA1-positive myeloid DCs (mDCs), BDCA3-positive mDCs, and plasmacytoid DCs (pDCs)—and determine their maturation markers (CD40, CD80, CD83, and CD86) in all participants. We also identified follicular DCs (fDCs), Langerhans DCs (LDCs), and pDCs in 42 patients by immunohistochemistry.

**Results:**

COPD was diagnosed in 43 patients (16 current smokers and 27 former smokers), whereas the remaining 32 subjects were classified as non-COPD (11 current smokers, 13 former smokers, and 8 never smokers). The number and maturation of DCs did not differ significantly between COPD and non-COPD patients. However, the results of flow cytometry indicated that maturation markers CD40 and CD83 of BDCA1-positive mDCs were significantly decreased in smokers than in non-smokers (P = 0.023 and 0.013, respectively). Immunohistochemistry also revealed a lower number of LDCs in COPD patients than in non-COPD subjects.

**Conclusions:**

Cigarette smoke, rather than airflow limitation, is the main determinant of impaired DCs maturation in the lung.

## Introduction

Chronic obstructive pulmonary disease (COPD) is a complex chronic inflammatory disease of the lungs characterized by progressive airflow limitation and destruction of the lung parenchyma.[1] Cigarette smoking is widely recognized as the main risk factor for COPD,[2] but less than 20% of all smokers will ultimately develop a clinically significant disease, suggesting an under-recognized contribution of additional genetic and environmental factors.[3]

Dendritic cells (DCs) are a heterogeneous population of antigen-presenting cells.[4] The classification of DCs in COPD remains controversial. Several research groups have identified different subsets of DCs in the human lung, mainly using antibodies raised against epitopes also present on circulating blood DCs, including Blood Dendritic Cell Antigen (BDCA) 1–3 and CD11.[5] A distinction between myeloid DCs (mDCs) and plasmocytoid DCs (pDCs) has been proposed. The mDCs subset can be further divided into the type 1 mDCs (mDCs1) and type 2 mDCs (mDCs2) subpopulations.[6] However, no data obtained by flow cytometry are currently available on pulmonary tissue resident mDCs, such as Langerhans-type DCs (LDCs) and follicular DCs (fDCs).[7]

The exact role of DCs in the pathogenesis of COPD is still a matter of debate, with human, animal, and *in vitro* investigations yielding conflicting results.[8] A study in a mouse model reported an increased number of lung DCs after exposure to cigarette smoke, suggesting their involvement in the inflammatory response elicited by tobacco.[9] Similarly, previous human studies demonstrated an increased number of DCs after tobacco exposure,[10] as well as their enhanced maturation and survival in COPD patients.[11] The number of DCs in lung tissues has been positively associated with both the presence[7, 11] and severity[12] of COPD. Moreover, an increased expression of maturation markers in lung DCs has been related to COPD severity.[13] At variance with these findings, another study reported a lower number of bronchial mucosal DCs in current smokers, with no significant differences between former smokers, non-smokers with asthma, and non-smoking controls.[14] Furthermore, other authors demonstrated a decreased maturation of DCs in the small airways and alveoli of COPD patients.[15] Importantly, Stoll and coworkers studied the surface molecules on bronchoalveolar lavage fluid DCs by flow cytometry in current smokers and former smokers with COPD and in never-smoking controls [16]. These authors found a significantly reduced expression of the maturation marker CD83 on myeloid DCs in current smokers with COPD and a strong reduction of chemokine receptor CCR5 on myeloid DCs in all patients with COPD, suggesting an impaired interaction with microbial agents. Additionally, Stoll and coworkers suggested that an imbalanced co-stimulation of DCs may contribute to the pathogenesis of COPD [17].

Starting from these premises, we designed the current study to investigate whether disease severity and smoking status could affect the distribution (BDCA1-positive mDCs, BDCA3-positive mDCs, and pDCs) and maturation (CD40, CD80, C83, and CD86) of DCs in lung tissues of patients undergoing elective pneumectomy or lobectomy for suspected primary lung cancer. To this aim, all of the study participants were analyzed according to the presence or absence of COPD and their smoking history.

## Methods

### Subjects

Consecutive patients in the surgical waiting list who were about to undergo elective pneumectomy or lobectomy for suspected primary lung cancer were identified upon the day of admission for elective surgery between December 2010 and July 2012. Patients who were <40 years of age, had an acute respiratory infection during the previous two months, had another previously diagnosed malignancy, received radiotherapy or chemotherapy, or suffered from other chronic inflammatory diseases were excluded from the study. Additionally, the time spent from the opening of the cutaneous layer through the extraction of the anatomical sample was measured, and cases in whom this time was >3 h were also excluded (since any potential stimulation of the inflammatory response due to surgery cannot be ruled out). The study population underwent a clinical evaluation by a pulmonologist. Patients whose spirometry results revealed a forced expiratory volume in the first second (FEV_1_)/forced vital capacity (FVC) ratio < 0.7 were considered as having COPD. All patients completed a standardized questionnaire recording their medical history, tobacco consumption, and actual treatments. The study followed the tenets of the Declaration of Helsinki and was approved by the Institutional Review Board of the Hospital Virgen del Rocío. All patients provided written informed consent prior to their inclusion in the study.

### Preparation of single cell suspensions from the lung and flow cytometry

Lung sections weighing approximately 5 g of parenchyma most distally located from the primary lesion were selected and immediately processed in our laboratory. The lung tissue samples were processed to obtain single cell suspensions for flow cytometry as described previously.[18] The lung tissue was minced with scissors and incubated with digestion medium in a humidified incubator at 37°C and 5% CO_2_ for 30 min. The medium was prepared using RPMI-1640 supplemented with 5% FBC, penicillin/streptomycin, L-glutamine, 2-mercaptoethanol, 1 mg/mL collagenase type 2 (Sigma; St. Louis, MO, USA) and 0.02 mg/mL DNase I (Sigma). Fresh digestion medium was then added and incubated for 15 min. The samples were centrifugated and resuspended in Ca^2+^- and Mg^2+^-free PBS containing 10 mM EDTA for 5 min at room temperature on a shaker. After passing through a 40-μm cell strainer (Becton Dickinson Labware; Bedford, MA, USA) with red blood cells being subjected to lysis, samples were washed and kept on ice until immunofluorescent labeling.

**Fig 1:**
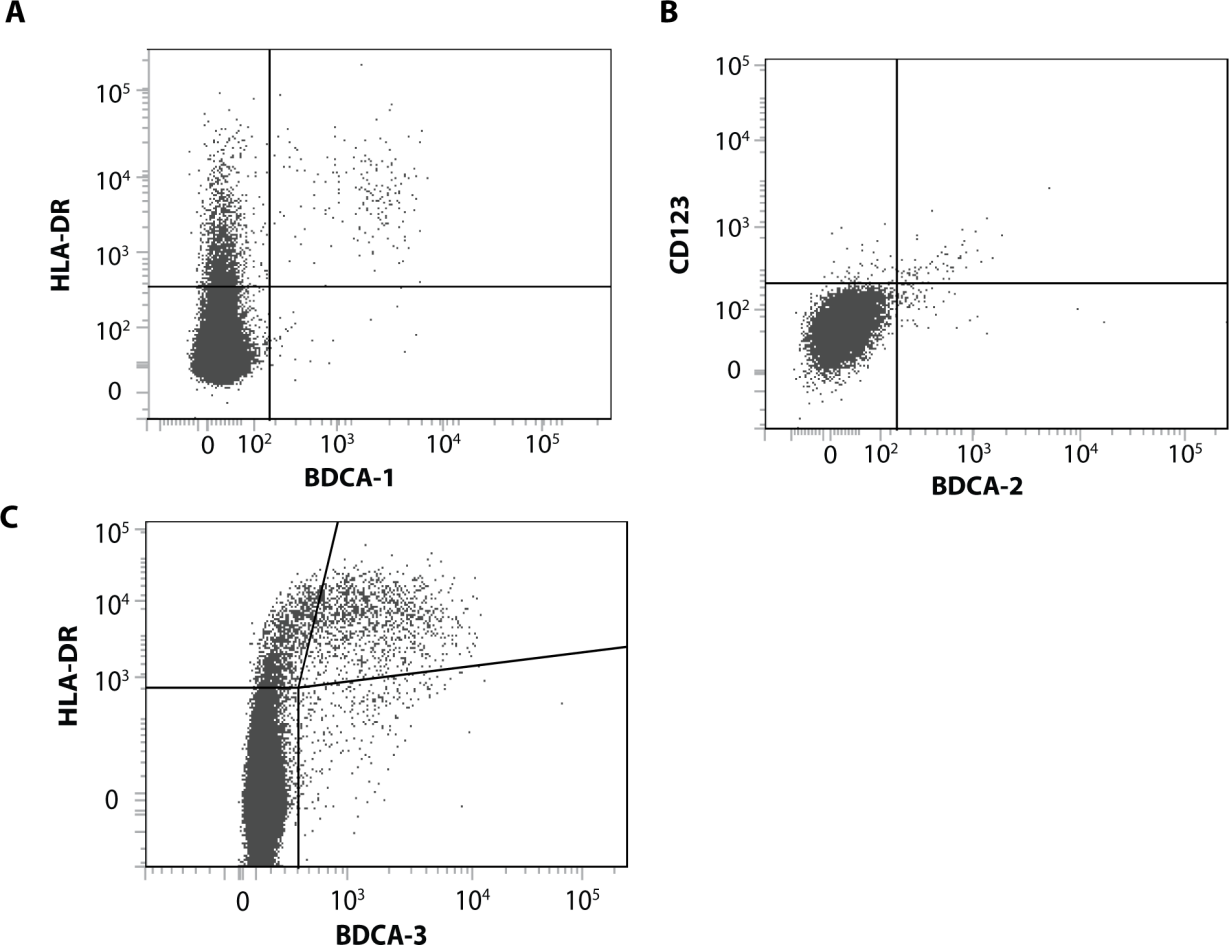
**Gating strategy for each subtype of DCs**: (A) BDCA1-positive mDCs; (B) BDCA3-positive mDCs; (C) pDCs.

DCs subsets were identified with flow cytometry performed on a Fortessa II (Becton Dickinson; Erembodegem, Belgium). After gating CD45+ cells characterized by a low autofluorescence, CD3+ and CD19+ cells were excluded to eliminate T cells, B cells, and alveolar macrophages. BDCA1+ and HLA-DR+ cells were considered as BDCA1-positive mDCs, whereas BDCA3-positive mDCs were defined as BDCA3+ and HLA-DR+ cells (Fig 1, panels A and B). pDCs were considered as being BDCA2+ and CD123+ (Fig 1, panel C). Results were expressed as the percentage of the total cell amount.

### Immunohistochemistry

Tissue blocks from the pulmonary parenchyma located distally from the primary lesion were fixed in 10% formalin and embedded in paraffin. Tissue sections (5-μm-thick) were de-paraffined with three changes of xilene (5 min each), hydrated with two changes of 100% ethanol (10 min each), and subsequently hydrated in 90% ethanol (5 min), ethanol 80% (5 min) and ethanol 70% (5 min). Antigen retrieval was performed in an automated fashion using PT Link (Dako; Carpinteria, CA, USA). The retrieval protocols utilized pre-specified settings in terms of preheating temperature, antigen retrieval temperature, time, and cool down. Antigen retrieval was generally performed at 97°C for 20 min. The bound antibody was developed with diaminobenzidine using a Dako Envision staining kit (K4065) according to the manufacturer’s recommendations. Two observers independently rated the stained sections under a light microscope. All of the immunohistochemical studies were performed using standard quality controls. Negative control experiments were performed using secondary antibodies in the absence of the primary antibody. Tissues were stained using mouse monoclonal antibodies raised against CD35, CD21, CD1a, CD68 (Dako) and Langerin (CD207) (Leica biosystem, United Kingdom). We identified DCs with morphology and specific markers. Specifically, CD21 and CD35 were used to identify fDCs,[19] whereas CD1a and CD207 were used to stain LDCs. CD68 was utilized for macrophages. Both fDCs and LDCs are CD1a+ and closely related to BDCA1-positive DCs.[7]

### Statistical analysis

Clinical variables are presented as means and standard deviations, medians, or absolute and relative frequencies, as appropriate. Intergroup differences were investigated with the Kruskal-Wallis test (continuous variables) or the χ^2^ test (categorical variables). Correlations between continuous variables were investigated using the Spearman’s rank correlation coefficient. One-way analysis of variance (ANOVA) was used to compare continuous variables between the four study groups, preceded by the Levene test of variance homogeneity. The Welch test was utilized when heterogeneity of variances was present. When probability values were < 0.05, the Mann–Whitney *U*-test was used to compare selected pairs of groups. All calculations were performed using the Statistical Package for the Social Sciences, version 20.0 (SPSS, IBM Corporation, Somers, NY, USA). Two-tailed p values < 0.05 were considered statistically significant.

## Results

### Patients and procedures

A total of 75 consecutive patients were included. COPD was diagnosed in 43 patients (16 current smokers and 27 former smokers), whereas the remaining 32 were classified as non-COPD (11 current smokers, 13 former smokers, and 8 never smokers). Table 1 shows the general characteristics of the study groups. Surgery consisted of pneumectomy, lobectomy, and atypical resections in 8.0%, 89.3%, and 2.7% of the study patients, respectively. Most operations (61.3%) were performed in the right hemithorax. A lung malignancy was diagnosed in 97.3% of the study participants. The most common histological types were adenocarcinomas (48%) and squamous cell carcinomas (37.3%).

**Table 1:** General characteristics of the study groups.

	COPD(n = 43)	Non-COPD, current smokers(n = 11)	Non-COPD, former smokers(n = 13)	Non-COPD, never smokers(n = 8)	P value*
Males, n (%)	40 (93.0)	8 (72.7)	13 (100)	6 (75.0)	0.070
Age, years	65.8 (7.7)	63.9 (11.3)	63.0 (12.4)	69.6 (7.0)	0.594
Tobacco history, pack-year)	61.7 (37.4)	55.9 (33.65)	49.9 (35.2)	0 (0)	< 0.001
FEV_1_, %	69.8 (16.7)	85.8 (18.1)	93.0 (12.1)	85.8 (18.1)	< 0.001
FEV_1_/FVC, %	58.5 (9.8)	75.7 (4.3)	74.3 (3.2)	73.1 (8.0)	< 0.001
LABA, n (%)	13 (31.0)	1 (9.1)	2 (15.4)	0 (0)	0.128
Inhaled steroids, n (%)	8 (19.0)	1 (9.1)	2 (15.4)	0 (0)	0.518
LAMA, n (%)	15 (35.7)	1 (9.1)	2 (15.4)	0 (0)	0.058
Oral steroids (n)	0 (0)	0 (0)	3 (23.1)	0 (0)	0.002

Data expressed as means (standard deviations) or absolute (relative) frequencies, as appropriate. FEV1: forced expiratory volume in one second; FVC: forced vital capacity; LABA: long-acting β_2_-agonists; LAMA: long-acting muscarinic antagonists.

*P values were calculated with the Kruskal–Wallis test for continuous variables and the χ^2^ test for categorical variables.

### Identification of different subtypes of lung dendritic cells

**Fig 2:**
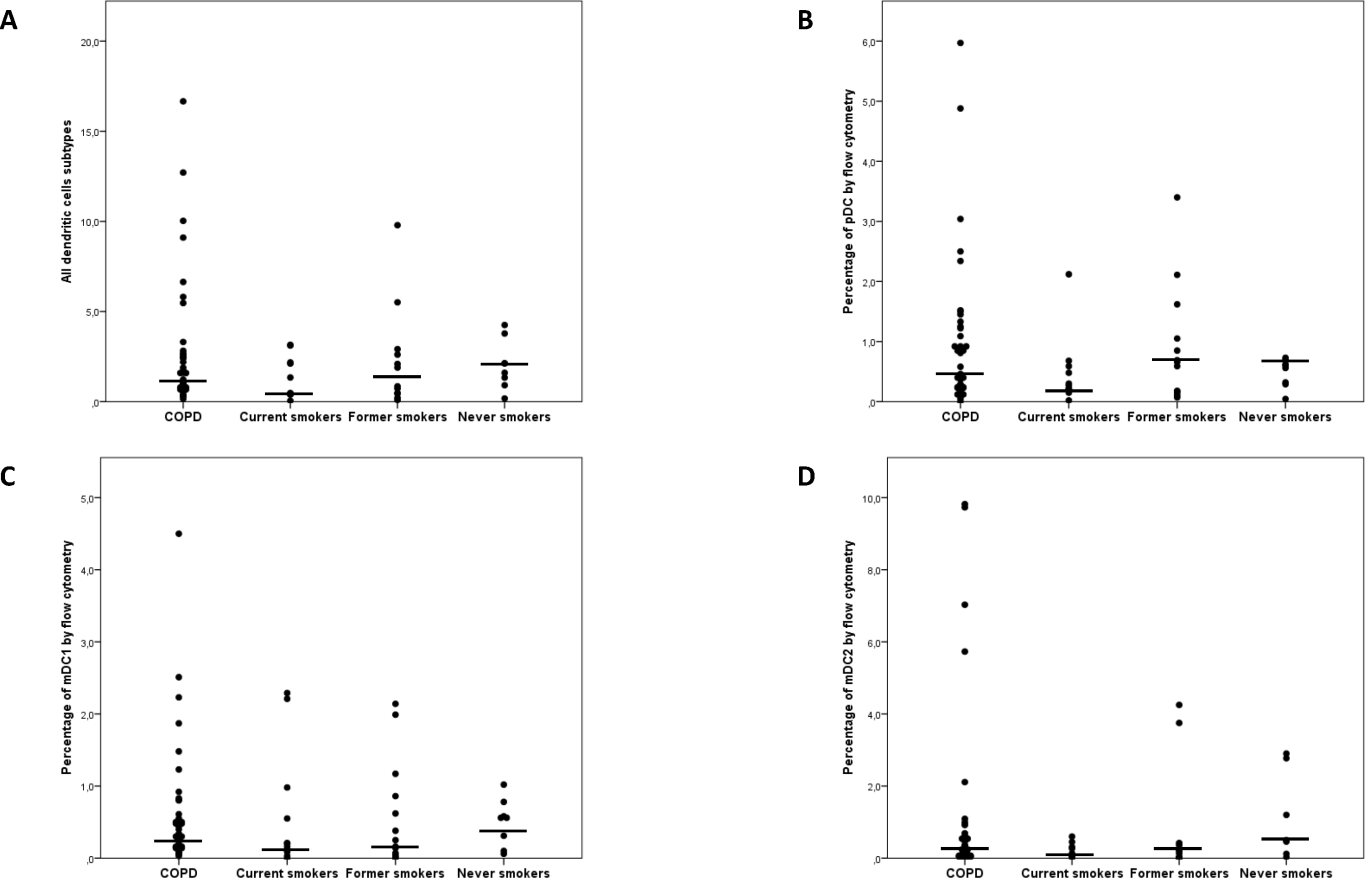
Distribution of dendritic cells and their subtypes in the study groups. No significant differences were identified.

In general, DCs were rarely identified in lung specimens. The percentage of DCs was <10% in all cases, with a mean value <1% for each subtype by flow cytometry. The most commonly identified subtypes of DCs in the entire study cohort were BDCA3-positive mDCs (0.90%), BDCA1-positive mDCs (0.81%), and pDCs (0.60%). The number of DCs did not differ significantly between COPD patients and non-COPD subjects (Fig 2). Interestingly, current smokers had a lower number of DCs (especially BDCA3-positive mDCs). COPD patients showed a higher expression of BDCA3-positive mDCs [1.09 (2.4)%] than BDCA2 [0.9 (1.2)%; P = 0.038], which was not observed in non-COPD subjects. The correlations between different subtypes of DCs varied according to the study subgroups (Table 2). Although highly significant positive correlations between mDCs subtypes were present in all groups, a positive association between myeloid and plasmacytoid subtypes was observed for COPD patients only.

**Table 2:** Correlations between different types of dendritic cells in the study groups.

	COPD(n = 43)	Non-COPD, current smokers(n = 11)	Non-COPD, former smokers(n = 13)	Non-COPD, never smokers(n = 8)
BDCA1^+^mDCs–BDCA3^+^mDCs	r = 0.559p < 0.001	r = 0.745p = 0.008	r = 0.832p = 0.001	r = 0.503p = NS
BDCA1^+^mDCs–pDCs	r = 0.417p = 0.005	r = 0.391p = NS	r = 0.286p = NS	r = 0.036p = NS
BDCA3^+^mDCs–pDCs	r = 0.386p = 0.013	r = 0.318p = NS	r = 0.538p = NS	r = 0.214p = NS

Correlations were calculated using the Spearman’s rank correlation coefficient.

**Fig 3:**
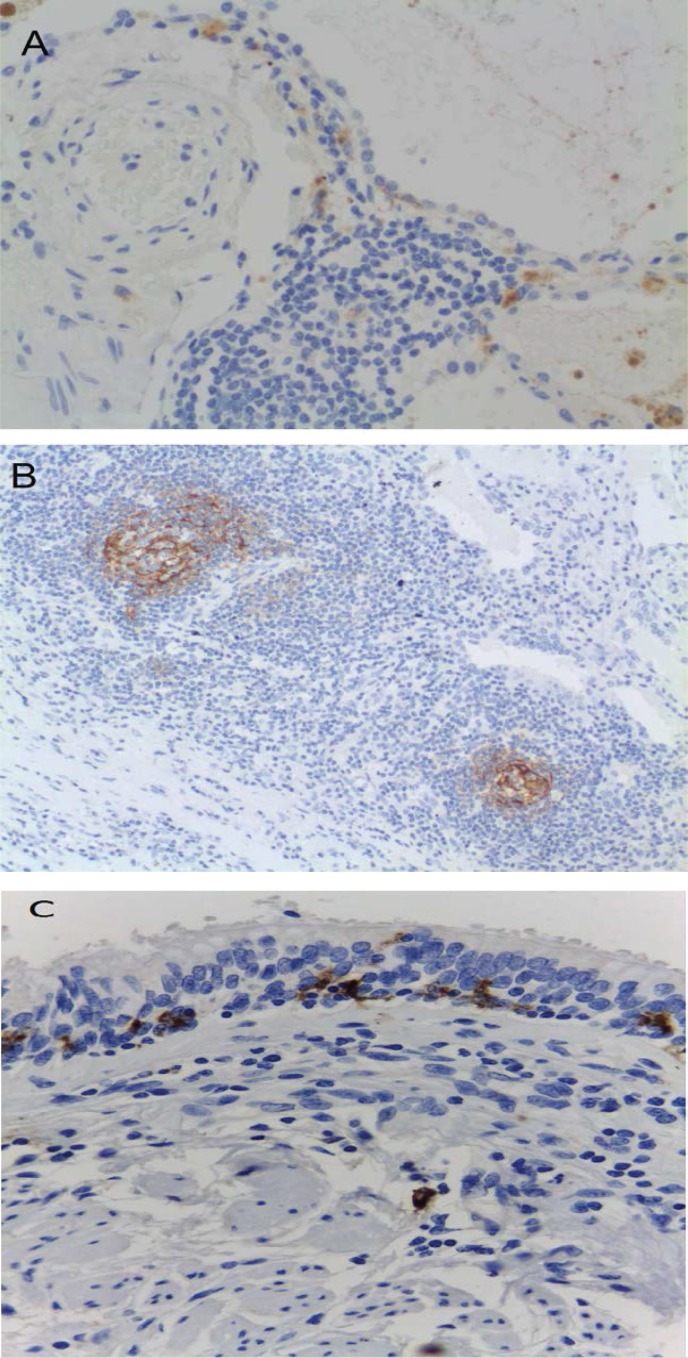
**Immunohistochemical staining:** CD1a (panel a), CD21 (panel b), and CD207 (panel c).

Immunohistochemistry was performed in 42 cases (27 COPD and 19 non-COPD). The results demonstrated the ubiquitous presence of macrophages in all specimens, whereas we failed to identify CD35+ cells. There was a trend toward a higher number of follicular DCs (CD21^+^) in the peribronchial lymphoid tissue of COPD patients compared with non-COPD subjects (22.2% *versus* 15.8%, respectively), although the difference failed to reach statistical significance (Fig 3A). Similarly, CD1a+ tended to be less frequent in COPD patients than in non-COPD subjects (33.3% *versus* 47.4%), albeit not significantly so. Most CD1a^+^ cells had a peribronchial location and showed a focal distribution. However, a few cases were characterized by a diffuse interstitial pattern (Fig 3B). The samples were also stained with CD207 (langerin; Fig 3C). The results demonstrated that the number of CD207^+^ cells tended to be less frequent in COPD patients (11%) than in non-COPD subjects (24%). In COPD patients, the CD207 marker was expressed in LDCs of the bronchial mucosa. However, 29% of the non-COPD subjects did not express the CD207 marker in the bronchial mucosa and interstice. A positive correlation between the number of CD1a+ and CD207+ cells was evident (r = 0.446, p = 0.006).

We found no correlation between the results of flow cytometry and immunohistochemistry. BDCA1-positive mDCs showed non-significant negative correlations with CD1a, CD21, CD35, and CD207 (r = -0.057, r = -0.045, r = -0.41 and r = -0.78, respectively; p>0.05). BDCA2-positive cells demonstrated non-significant positive correlations with CD1a, DC21, CD35, and CD207 (r = 0.076, r = 0.087, r = 0.07, and r = 0.103, respectively; p>0.05). BDCA3-positive cells showed non-significant negative correlations with CD1a, CD21, and CD35 (r = -0.203, r = -0.154 and r = -0.175, respectively; p>0.05). Eleven patients were receiving a fixed combination of an inhaled corticosteroid (ICS) plus a long-acting β_2_ agonist (LABA). We failed to identify significant differences in the number of different DCs according to the use of combination treatment.

### Markers of dendritic cell maturation

**Fig 4:**
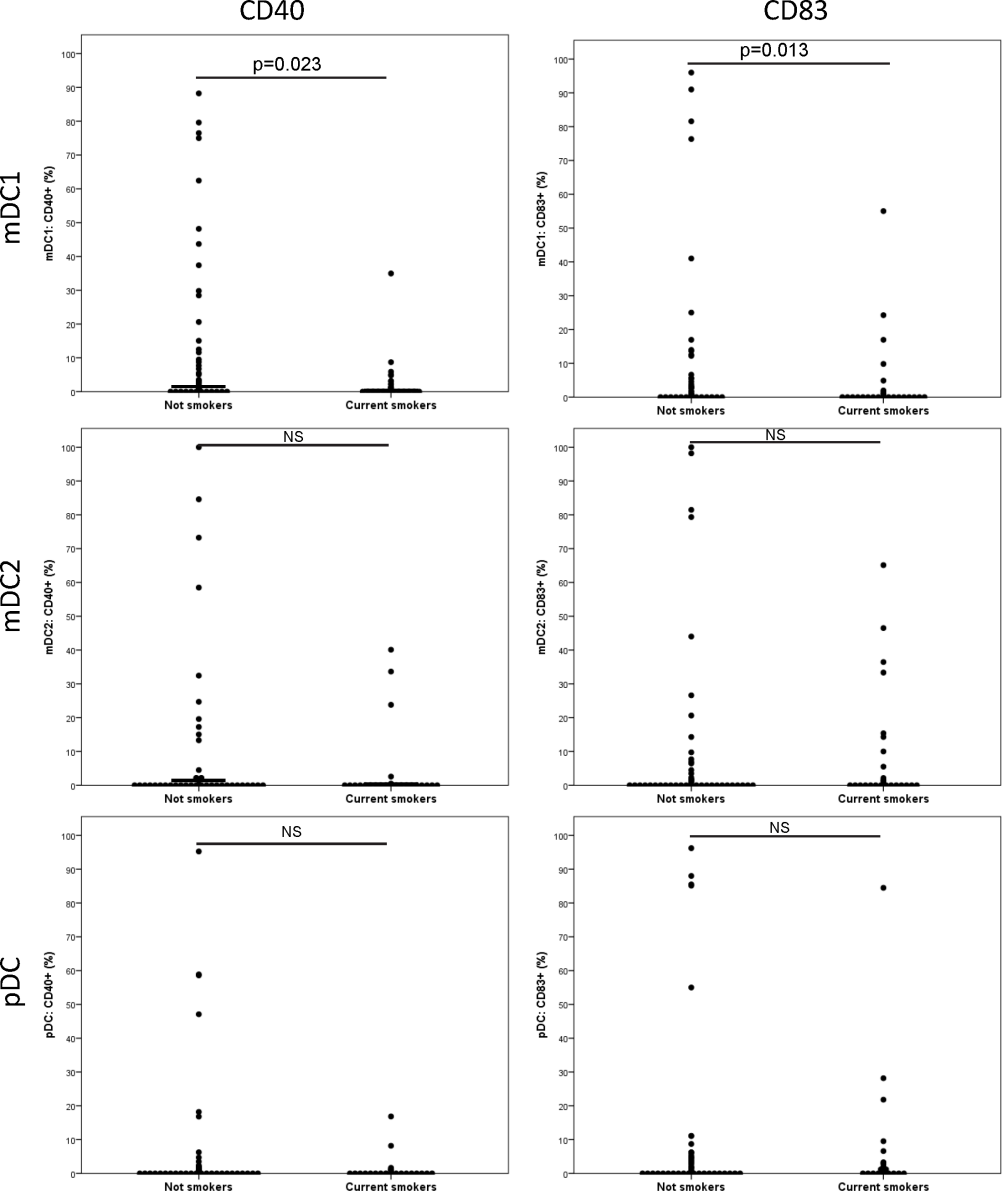
Expression (assessed by flow cytometry) of the maturation markers CD40 and CD83 in different dendritic cell subtypes according to the smoking status.

We found that both BDCA1-positive and BDCA3-positive mDCs expressed higher levels of CD40 than pDCs (p = 0.009 and p = 0.065, respectively). In addition, both BDCA1-positive and BDCA3-positive mDCs were characterized by a higher expression of CD86 than pDCs (p = 0.001 and p = 0.012, respectively). Maturation markers did not differ significantly between COPD patients and non-COPD subjects. However, significant differences were evident between current smokers and former smokers, regardless of the presence of COPD (Fig 4). Current smokers showed a decreased expression of DCs maturation markers, including CD40 (p = 0.023) and CD83 (p = 0.013) for BDCA1-positive mDCs. Within COPD patients, active smokers similarly showed a lower percentage of DCs expressing maturation markers (Table 3). We did not find significant differences in the expression of maturation markers according to the use of combination treatment (ICS plus LABA).

**Table 3:** Subtypes and maturation markers (assessed by flow cytometry) of dendritic cells (expressed as percentages) in current and former smokers with COPD.

DC subtype	Maturationmarker	COPD, current smokers(n = 16)	COPD, former smokers(n = 27)	P value*
BDCA1^+^-mDCs	CD40	3.7 (8.6)	14.3 (23.0)	0.039
	CD80	8.2 (15.9)	10.3 (15.3)	NS
	CD83	6.1 (14.4)	10.3 (23.7)	NS
	CD86	15.3 (22.5)	24.4 (26.8)	NS
BDCA3^+^-mDCs	CD40	4.2 (11.2)	13.5 (27.3)	NS
	CD80	8.6 (19.9)	11.9 (28.1)	NS
	CD83	10.6 (18.8)	15.1 (32.1)	NS
	CD86	21.3 (22.1)	21.5 (23.5)	NS
pDCs	CD40	1.2 (4.1)	6.0 (14.6)	NS
	CD80	11.4 (17.2)	12.4 (15.5)	NS
	CD83	8.4 (21.0)	12.1 (28.1)	NS
	CD86	13.5 (13.7)	13.4 (17.8)	NS

* Mann–Whitney *U*-test. NS: not significant.

Also, we have compared the maturation markers of dendritic cells in current and former smoker non-COPD and we have found differences in both groups. The former smokers express more maturation markers than current smokers. These differences are significant for BDCA1^+^ mDCs-CD40^+^ (p = 0.036), BDCA1^+^ mDCs-CD86+ (p = 0.011) and BDCA3^+^ mDCs-CD86 (p = 0.034) (Table 4).

**Table 4:** Subtypes and maturation markers of dendritic cells (expressed as percentages) in current and former smokers non-COPD by flow cytometry.

DC subtype	Maturationmarker	Current smokers(n = 11)	Former smokers(n = 13)	P value*
BDCA1^+^ mDCs	CD40	1.3 (1.9)	20.8 (32.0)	0.036
	CD80	13.4 (22.0)	15.3 (23.4)	NS
	CD83	1.6 (5.0)	17.3 (30.3)	NS
	CD86	13.0 (15.4)	14.3 (19.3)	0.011
BDCA3^+^ mDCS	CD40	3.1 (10.12)	13.5 (29.3)	NS
	CD80	7.8 (24.1)	15.4 (25.2)	NS
	CD83	5.8 (13.9)	13.3 (27.9)	NS
	CD86	3.0 (9.1)	20.5 (32.9)	0.034
pDCs	CD40	0.9 (2.42)	11.4 (28.7)	NS
	CD80	10.8 (19.1)	13.4 (25.8)	NS
	CD83	3.2 (8.3)	17.2 (32.8)	NS
	CD86	7.3 (10.9)	7.5 (12.5)	NS

* Mann–Whitney *U*-test. N/A: not applicable; NS: not significant

When current smokers with COPD were compared with non-COPD subjects, no significant differences were found in terms of subtypes and maturation markers of dendritic cells (Table 5). Similarly, former smokers with COPD did not differ from non-COPD subjects (data not shown).

**Table 5:** Subtypes and maturation markers of dendritic cells (expressed as percentages) in current smokers with COPD and control group.

DC subtype	Maturationmarker	COPD, current smokers(n = 16)	Control group(n = 32)	P value*
BDCA1-positive mDCs	CD40	4.1 (9.2)	9.1 (20.0)	NS
	CD80	8.0 (17.0)	11.9 (20.1)	NS
	CD83	5.3 (14.5)	6.7 (16.0)	NS
	CD86	11.4 (15.2)	21.9 (25.4)	NS
BDCA3-positive mDCs	CD40	4.6 (12.0)	7.4 (20.8)	NS
	CD80	9.8 (21.1)	11.0 (21.4)	NS
	CD83	11 (19.9)	7.1 (19.6)	NS
	CD86	21.5 (23.5)	13.5 (23.2)	NS
pDCs	CD40	1.3 (4.4)	7.4 (21.8)	NS
	CD80	11.8 (18.5)	16.0 (24.2)	NS
	CD83	9.5 (22.3)	7.17 (21.7)	NS
	CD86	10.5 (11.9)	8.3 (11.3)	NS

### Association with disease severity

**Fig 5:**
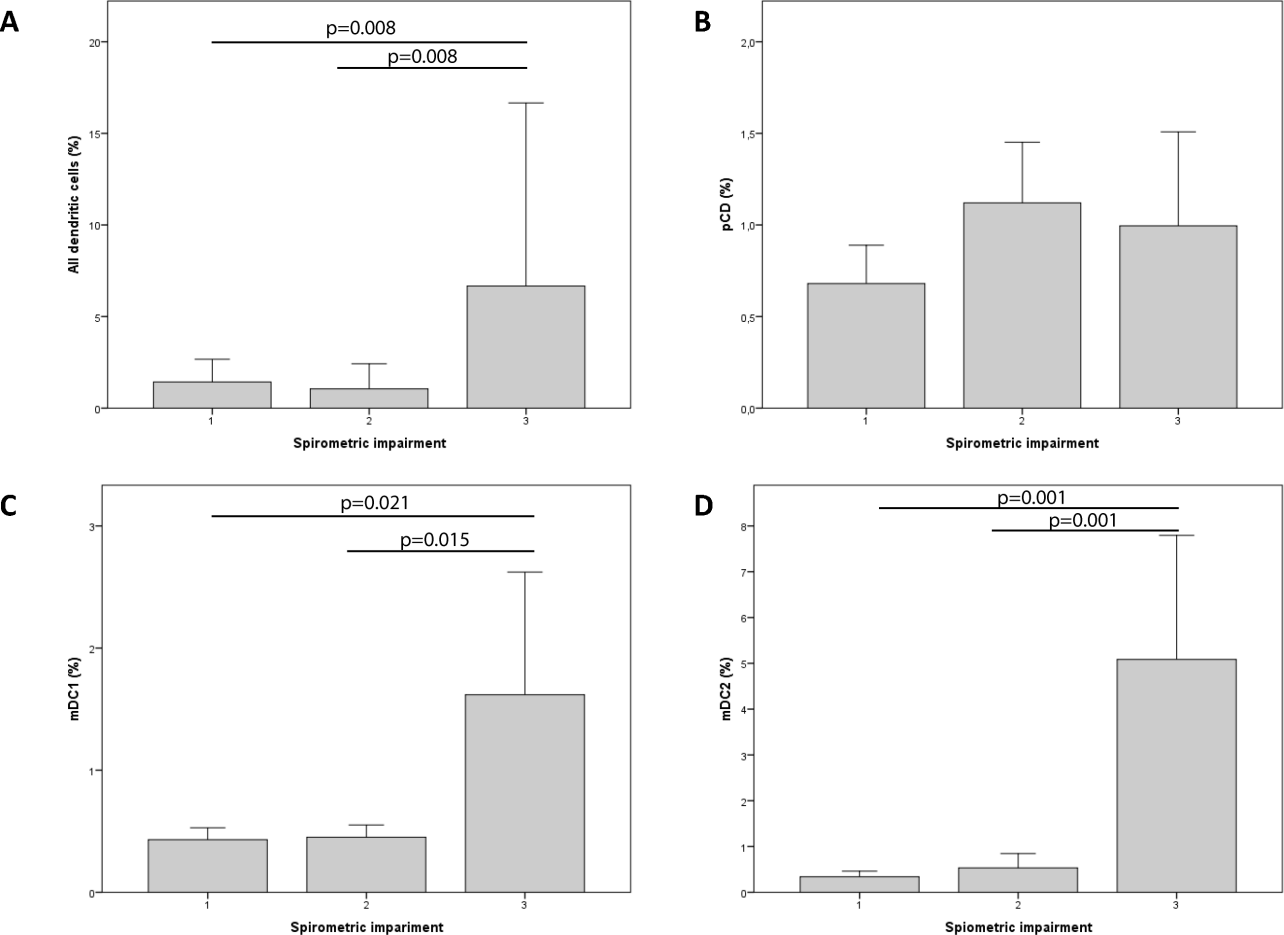
Associations between disease severity (expressed by spirometric impairment) and the percentage of dendritic cells measured by flow cytometry.

Because data on the last spirometry were missing for 5 COPD cases, the analysis of COPD severity was limited to 38 COPD patients. The number of mDCs (BDCA1- or BDCA3-positive) was significantly associated with disease severity as measured by the spirometric impairment. Specifically, patients with severe disease (stage 3) showed a higher number of DCs (Fig 5). Interestingly, maturation markers were more frequently expressed in patients with stages 1−2 COPD compared with stage 3, the only exception being CD86 (Table 6).

**Table 6:** Subtypes and maturation markers of dendritic cells (expressed as percentage) in different stages of COPD (stage 3 *versus* stages 1−2).

DC subtype	Maturation marker	COPD, stages 1−2(n = 34)	COPD, stage 3(n = 4)	P value*
BDCA1^+^mDCs	CD40	11.1 (19.5)	0.7 (0.9)	0.004
	CD80	10.4 (16.0)	2.1 (0.8)	0.005
	CD83	10.1 (22.9)	3.5 (6.03)	NS
	CD86	20.5 (24.2)	21.1 (27.3)	NS
BDCA3^+^mDCs	CD40	10.7 (23.8)	0.4 (0.8)	0.021
	CD80	10.8 (23.9)	0 (0)	N/A
	CD83	14.3 (28.3)	0.5 (0.6)	0.008
	CD86	20.6 (21.8)	22.2 (29.1)	NS
pDCs	CD40	3.8 (11.06)	0.6 (0.9)	NS
	CD80	11.5 (15.1)	6.2 (12.5)	NS
	CD83	12.6 (14.1)	11.2 (28.4)	0.048
	CD86	12.02 (14.1)	11.2 (15.3)	NS

*Welch test. N/A: not applicable; NS: not significant.

## Discussion

The inflammatory response occurring in COPD involves the participation of multiple cell types (e.g., neutrophils, monocytes/macrophages, B and T lymphocytes, and DCs), but their exact role in airway injury and remodeling is not yet entirely elucidated. Moreover, the question as to whether DCs can mediate the inflammatory reaction elicited by cigarette smoke remains open. In this study, we used flow cytometry and immunochemistry to investigate both the distribution and maturation markers of DCs in the lung parenchyma. Our main results are as follows: 1) the number of all DCs subtypes measured by flow cytometry did not differ significantly between COPD patients and non-COPD subjects; 2) current smokers had a reduced number of DCs (especially BDCA3-positive mDCs2); 3) the correlations between different subtypes of DCs varied according to the study subgroups; and 4) current smoking was associated with a reduced expression of maturation markers in DCs. In general, immunohistochemistry revealed a peribronchial distribution of DCs with minor intergroup differences.

Although several previous reports have investigated the role of lung DCs in the pathogenesis of COPD, the results have been conflicting. Using immunohistochemistry, Demedts *et al*.[20] identified a higher number of DCs in the bronchial tissues of COPD patients compared with controls; notably, the number of DCs was positively associated with the severity of COPD. Rogers and coworkers[14] examined by electronic microscopy the bronchial biopsies of 37 subjects (15 patients with COPD, 11 patients with asthma, and 11 controls) and observed a decreased number of DCs in COPD patients. Using flow cytometry of lung leukocytes, Freeman and colleagues[13] found no significant differences in the number of DCs between COPD patients and healthy controls. Van Pottelberge *et al*.[7] demonstrated that LDCs in the lung are closely related to mDCs1 (defined by the presence of BDCA1). The authors reported an increased number of LDCs in COPD, which showed a positive association with disease severity. In contrast, BDCA1-positive DCs (which are precursors of LDCs)[7] were significantly under-represented in COPD patients and decreased in parallel with disease severity. The authors inferred the presence of an altered DCs differentiation in COPD, leading to a selective accumulation of LDCs accompanied by a corresponding decrease in their BDCA1-positive precursors.[12] Subsequently, the same research group investigated the distribution of pDCs in the lungs of 74 subjects (32 controls, 28 patients with stage I−II COPD, and 14 patients with stage III−IV COPD).[21] The results indicated that pDCs were mainly located in the follicles, with a higher number being evident in COPD patients compared with controls. The number of lung pDCs increased in parallel with the severity of the disease. However, no significant differences were observed in the number of pDCs in the airways. Stoll *et al*.[17] did not find significant differences in total mDCs between COPD patients and healthy controls. Our results indicate that the number of DCs did not differ significantly between COPD patients and non-COPD subjects, regardless of the technique used for their detection (immunohistochemistry or flow cytometry). In general, the interpretation of the results obtained with different methodologies should be performed with caution. In our study, the results of flow cytometry indicated a higher number of DCs in COPD patients, but a lower number of CD1a- and CD207-positive cells. It is noteworthy that different markers were used in immunohistochemistry and flow cytometry studies.

There were no significant differences in the number and maturation of DCs in patients treated with ICS plus LABA *versus* those who received ICS or LABA alone. Lommatzsch et al.[22] reported that a combination therapy with fluticasone plus salmeterol was associated with a reduction in airway mDCs (BDCA1-positive and BDCA3-positive cells). Despite the apparent contradiction with our current findings, it should be noted that the populations under investigation and the methodologies used were different. First, Lommatzsch et al.[21] conducted their analysis using bronchoalveolar fluid and blood. In addition, patients with an oxygen saturation <90%, FEV1<80%, and chronic disorders (the only exception being arterial hypertension) were excluded.

In line with previous data,[10, 13, 20, 21] we did not find a significant impact of the smoking status on the number of lung DCs. Stoll at al.[17] reported an imbalanced expression of co-stimulatory molecules on blood DCs of COPD patients. The authors reported that the altered expression of CD83 on blood DCs was related to smoking, suggesting its potential role in the pathogenesis of the disease. Our results indicate that the maturation markers CD40, CD80, and CD83 of BDCA1-positive mDCs are significantly decreased in smokers compared with non-smokers. These findings are in accordance with the recent study by Liao and coworkers.[23] In a sample of 32 subjects (8 smokers without COPD, 8 smokers with COPD, 8 non-smokers without COPD, and 8 non-smokers with COPD), the authors reported a decreased expression of CD83 (as assessed by reverse transcription quantitative polymerase chain reaction) in lung mDCs of smokers compared with those of non-smokers. A similar decrease in sputum mature DCs has been described in healthy smokers and patients with COPD.[24] Givi et al.[25] have recently demonstrated that cigarette smoke modulates the maturation and function of primary murine DCs. Our results confirm and expand their results using human lung tissue of COPD patients and non-COPD subjects.

Differently from previous studies showing a higher number of mature DCs in severe COPD,[11, 13, 21] we were unable to find significant differences in terms of maturation markers between COPD patients and non-COPD subjects. Differences in patient characteristics − including the use of medications − may at least in part explain the observed discrepancies. In this regard, the impact of inhaled corticosteroids on the number and maturation of DCs remains controversial. It has been reported that corticosteroids may decrease the number of BDCA1-positive mDCs[26, 27] mainly via induction of apoptosis [4, 28, 29]. However, other authors were unable to identify an association between the use of inhaled corticosteroids and the number of DCs,[9] being in accordance with our current data.

In summary, the current study confirms previous results from the work of Stoll et al.[16] by showing that cigarette smoke, rather than airflow limitation, is the main determinant of impaired DCs maturation in the lung. Further research is needed to investigate the contribution of an altered DCs maturation to the pathogenesis of tobacco-related diseases.
